# Experience of the concentration of methotrexate evaluation in children with juvenile idiopathic arthritis

**DOI:** 10.1186/1546-0096-9-S1-P187

**Published:** 2011-09-14

**Authors:** FV Rokhlina, GA Novik, MF Dubko

**Affiliations:** 1 The State Medical Pediatric Academy of Saint-Petersburg, Russia

## 

Juvenile idiopathic arthritis - one of the most common rheumatic disease in children (1:1000) [Timothy Beukelman et all, 2011]. The most effective basic drug is methotrexate, an initial dose of 15 mg/m2/week. Our study to determine the concentration of methotrexate in serum aims to develop a test system to clarify the reasons for lack of efficacy of methotrexate in some patients. The study currently includes 46 children (19 boys (41,3%), 27 girls (58.7%)) with different forms of JIA, according to the classification of ILAR. Systemic arthritis – 9 children (20%), arthritis oligoarticular – 9 (20%), polyarticular arthritis – 15 (34%), psoriatic arthritis – 1 (2%), arthritis entesitis – 11 (24%). Of the 46 children noone is in clinical remission off medication. 26 (56.5%) children met the criteria of inactive disease.

Results of determining the concentration of methotrexate in children with JIA were distributed as follows - 0-1 umol/l - 2,18%; 1-1,5 umol/l - 34.78%; 1,5-2 umol/l - 17.39 % 2-2,5 umol/l - 32.61%; 2,5-3 umol/l - 8.69% above 3 umol/l - 4,35%. Among children with the results of the concentration of methotrexate up to 1.5 umol/l - 88.24% have signs of active disease (elevated ESR / CRP high / dysproteinemia / active joints). Low concentrations of methotrexate in 73.3% are associated with high levels of ESR and CRP. Among children with the results of methotrexate concentration above 1,5 umol/l signs of active disease manifestations are much rarer in 48,28% of cases.

